# Efficacy of ground herb-based and essential oil-based phytobiotics on the intestinal health and performance of nursery pigs challenged with F18^+^*Escherichia coli*

**DOI:** 10.1093/jas/skaf018

**Published:** 2025-01-31

**Authors:** Yesid Garavito-Duarte, Marcos Elias Duarte, Sung Woo Kim

**Affiliations:** Department of Animal Science, North Carolina State University, Raleigh, NC 27695, USA; Department of Animal Science, North Carolina State University, Raleigh, NC 27695, USA; Department of Animal Science, North Carolina State University, Raleigh, NC 27695, USA

**Keywords:** F18^+^*Escherichia coli*, intestinal health, phytobiotics, oxidative stress, nursery pigs

## Abstract

This study aimed to evaluate the efficacy of using ground herb-based phytobiotics and essential oil-based phytobiotics in pig diets on intestinal health and growth performance (**GP**) of nursery pigs challenged with F18^+^*Escherichia coli*. Forty nursery pigs (6.4 ± 0.1 kg) at 21 d of age were individually housed and assigned to 4 dietary treatments in a randomized complete block design, with body weight and sex as blocking factors. Basal diets were fed to pigs for 28 d in 3 phases. Treatments were negative control (**NC**): basal diet, non-challenged; positive control (**PC**): basal diet, challenged with F18^+^*E. coli*; HP: PC + 1% ground herb-based phytobiotics (Salcochek Pro, Ayurvet Limited, Kaushambi, India); EP: PC + 1% essential oil-based phytobiotics (Liq-biotic, Ayurvet Limited). The GP was recorded for each phase and fecal score (**FS**) was measured daily. On day 7 postweaning, the challenged groups were orally inoculated with F18^+^*E. coli* (2.0 × 10^10^ CFU), the NC treatment received a sterile saline solution. On day 28, pigs were euthanized to collect jejunal samples to evaluate intestinal health and relative abundance (**RA**) of jejunal mucosa-associated microbiota. Data were analyzed using the MIXED procedure on SAS 9.4. The PC increased (*P* < 0.05) the RA of Prevotellaceae, Lachnospiraceae, and Ruminococcaceae when compared to NC. The HP reduced (*P* < 0.05) the RA of Veillonellaceae, Prevotellaceae, and Lachnospiraceae when compared to PC. The EP tended to reduce the RA of Streptococcaceae (*P* = 0.073) and Corynebacteriaceae (*P* = 0.074) when compared to PC. The PC increased (*P* < 0.05) occludin and tended to increase (*P* = 0.096) toll-like receptor-4 (**TLR4**) when compared to NC. The PC decreased (*P* < 0.05) average daily gain and average daily feed intake when compared to NC in days 7 to 28. The PC increased FS (P < 0.05) compared to the HP and EP days 7 to 11. The HP and EP decreased (*P* < 0.05) FS when compared to PC during days 7 to 11 and days 7 to 18. In conclusion, F18^+^*E. coli* challenge disrupted the jejunal mucosa-associated microbiota, increased TLR4 expression and FS, and consequently reduced GP. Both HP and EP phytobiotics supported intestinal morphology during the challenge to F18^+^*E. coli* by supporting enterocyte maturation. The HP and EP treatments exhibited antimicrobial-like effects by altering the jejunal mucosa-associated microbiota and reduced FS during the first 2 weeks post-challenge. The HP treatment showed potential antioxidant effects.

## Teaser

Ground herb-based and essential oil-based phytobiotics supported intestinal morphology in nursery pigs challenged with F18^+^*E. coli*. Both phytobiotics exhibited antimicrobial properties and reduced fecal scores, offering promising insights into swine nutrition and disease management.

## Introduction

Postweaning diarrhea (**PWD**) frequently occurs due to the stress generated during the weaning period, predisposing pigs to enterotoxigenic *Escherichia coli* infection. Enterotoxigenic *E. coli* expressing F18 fimbriae and producing heat-stable toxin A (Sta) and heat-stable toxin B (STb) are the primary causes of diarrhea in nursery pigs (Liu et al., 2014; Castro et al., 2022). Generally, PWD occurs between the first and second weeks postweaning, causing watery diarrhea with potential mortality rates between 1.5% and 25% (Duarte et al., 2023). The repercussions of such infections extend beyond the gastrointestinal tract, including a diminished growth rate, and substantial economic losses within pig production systems (Coddens et al., 2017).

The optimal performance of pigs is highly linked to maintaining optimal intestinal conditions. The small intestine is not only a critical site for nutrient digestion and absorption but also the largest component of the animal’s immune system (Chassaing et al., 2014). Among the nutritional strategies explored to promote intestinal health, phytobiotics, plant-derived additives with bioactive compounds, have gained attention for their potential to enhance growth performance (GP) and intestinal health (Kommera et al., 2006; Kikusato, 2021).

Phytobiotics involve various plant-based sources, including ground herbs and essential oils, which provide unique combinations of bioactive compounds with functional properties. Ground herbs, such as *Aegle marmelos*, *Berberis aristata*, *Holarrhena antidysenterica*, and *Punica granatum*, are rich in coumarins, flavonoids, saponins, steroid alkaloids, and tannins. These compounds exhibit antidiarrheal (Gilani et al., 2010; Rahman and Parvin, 2014; Sharma et al., 2015), antimicrobial (Sinha et al., 2013; Mujeeb et al., 2014; Jahan et al., 2022), anti-inflammatory (Raj et al., 2020), and antioxidant (Lamichhane et al., 2014; Bassiri-Jahromi, 2018) properties. Similarly, essential oils from plants like *Eucalyptus globulus*, *Mentha piperita*, *Ocimum sanctum*, and *Trachyspermum ammi* are composed of eucalyptol, menthol, thymol, and other phenolic compounds with antimicrobial and antioxidant activities (Bairwa et al., 2012; Yamani et al., 2016; Čmiková et al., 2023).

Evaluating the effects of ground herb-based phytobiotics and essential oil-based phytobiotics could provide new insights directly into the modulation of jejunal mucosa-associated microbiota to improve intestinal health and in nursery pigs challenged with F18^+^*E. coli*. In the present study, it was hypothesized that the dietary inclusion of ground herb-based phytobiotics could reduce oxidative stress, support intestinal barrier integrity, and improve fecal score (**FS**), thereby regulating inflammatory responses. Additionally, essential oil-based phytobiotics will increase beneficial bacteria in the jejunal mucosa reducing jejunal oxidative stress, collectively mitigating the detrimental effects of F18^+^*E. coli* in the jejunum of nursery pigs. The objective of this study was to evaluate the efficacy of dietary inclusion of ground herb-based phytobiotics and essential oil-based phytobiotics on the diversity and relative abundance (**RA**) of jejunal mucosa-associated microbiota and mucosal immune response in the jejunum of nursery pigs challenged with F18^+^*E. coli*.

## Materials and Methods

The experimental protocol was approved by the Institutional Animal Care and Use Committee of North Carolina State University. Two different phytobiotics were used in this study (Table 1) 1) ground herb-based phytobiotics (Salcochek Pro, Ayurvet Limited, Kaushambi, India) mainly composed of *A. marmelos* (14.4%), *Berberis aristate* (14.4%), *H. antidysenterica* (14.4%), *P. granatum* (14.4%), and *Woodfordia fruticose* (14.4%); and 2) essential oil-based phytobiotics (Liq-biotic, Ayurvet Limited) mainly composed of moisture (98.3 %), *M. piperita* (0.75%), *E. globulus* (0.65%), *O. sanctum* (0.05), and *Trychyspermum ammi* (0.05%).

**Table 1.** Composition of ground herb-based phytobiotics and essential oil-based phytobiotics

Item	Component	Content, %
Ground herb-based phytobiotics^1^	*Holarrhena antidysenterica*	14.4
	*Berberis aristate*	14.4
	*Punica granatum*	14.4
	*Aegle marmelos*	14.4
	*Woodfordia fruticose*	14.4
	Others	28.0
Essential oil-based phytobiotics^2^	Moisture	96.3
	*Ocimum sanctum*	0.05
	*Mentha piperita*	0.75
	*Eucalyptus globulus*	0.65
	*Trychyspermum ammi*	0.05
	Others	2.20

^1^Salcochek Pro (Ayurvet Limited).

^2^Liq-biotic (Ayurvet Limited).

### Animals, design, and diets

The experiment was conducted at the Metabolism Education Unit at North Carolina State University (Raleigh, NC). Forty pigs (PIC 337 × Camborough 22) at 21 d of age (20 barrows and 20 gilts) with initial body weight (**BW**) of 6.4 ± 0.1 kg were allotted to 4 dietary treatments based on a randomized complete block design with sex and BW as a block. The basal diets were composed of a corn and soybean meal-based formulation. The dietary treatments consisted of a negative control (**NC**): basal diet, non-challenged pigs; positive control (**PC**): basal diet, challenged with F18^+^*E. coli* on day 7; test 1 diet (HP): basal diet + 1% ground herb-based phytobiotics (Salcochek Pro, Ayurvet Limited), incorporated into the feed during the mixing process, and challenged with F18^+^*E. coli* on day 7; and test 2 diet (EP): basal diet + 1% essential oil-based phytobiotics (Liq-biotic, Ayurvet Limited), incorporated into the feed during the mixing process, and challenged with F18^+^*E. coli* on day 7 postweaning. The inclusion level (1%) of both phytobiotics was decided to provide 5 g phytobiotics per day based on preliminary titration studies (Patilba et al., 2017). The phytobiotics were supplemented replacing corn in the basal diet and diets were formulated meeting the nutrient requirements of NRC (2012), as shown in Table 2. Pigs were housed individually with free access to feed and water during 28 d feeding period (4 wk) based on 3 phase feeding program: phase 1 (days 0 to 11), phase 2 (days 11 to 21), and phase 3 (days 21 to 28).

**Table 2.** Composition of basal diets (as-fed basis)

Item	Phase 1	Phase 2	Phase 3
Feedstuff, %			
Corn, yellow	41.96	49.52	60.57
Whey permeate, 80% lactose	19.00	13.00	6.00
Soybean meal, 48% CP	18.50	23.50	28.50
Poultry meal	9.00	5.00	0.00
Fish meal	5.00	3.00	0.00
Enzyme treated SBM^1^	3.00	1.50	0.00
Poultry fat	1.10	1.70	1.42
l-Lys HCl	0.58	0.47	0.46
l-Met	0.27	0.19	0.16
l-Thr	0.20	0.14	0.14
l-Trp	0.03	0.01	0.00
l-Val	0.02	0.01	0.03
Dicalcium phosphate	0.00	0.38	0.95
Limestone	0.44	0.68	0.87
Salt	0.22	0.22	0.22
Vitamin premix^2^	0.03	0.03	0.03
Mineral premix^3^	0.15	0.15	0.15
Calculated composition			
Dry matter, %	90.8	90.3	89.5
ME, kcal/kg	3,401	3,400	3,351
SID^4^ Lys, %	1.50	1.35	1.23
SID Met + Cys, %	0.82	0.74	0.68
SID Trp, %	0.25	0.22	0.20
SID Thr, %	0.88	0.79	0.73
SID Val, %	0.95	0.87	0.78
Ca, %	0.85	0.80	0.70
STTD P^5^, %	0.45	0.40	0.33
Total P, %	0.70	0.64	0.58

^1^HP300 (Hamlet Protein, Findlay, OH, USA).

^2^The vitamin premix provided the following per kilogram of complete diet: 6,613.8 IU of vitamin A as vitamin A acetate, 992.0 IU of vitamin D3, 19.8 IU of vitamin E, 2.64 mg of vitamin K as menadione sodium bisulfate, 0.03 mg of vitamin B12, 4.63 mg of riboflavin, 18.52 mg of d-pantothenic acid as calcium pantothenate, 24.96 mg of niacin, and 0.07 mg of biotin.

^3^The trace mineral premix provided the following per kilogram of complete diet: 4.0 mg of Mn as manganous oxide, 165 mg of Fe as ferrous sulfate, 165 mg of Zn as zinc sulfate, 16.5 mg of Cu as copper sulfate, 0.30 mg of I as ethylenediamine di-hydroiodide, and 0.30 mg of Se as sodium selenite.

^4^Standardized ileal digestible.

^5^Standardized total tract digestible phosphorus.

On d 7 postweaning, 30 pigs were orally inoculated with F18^+^*E. coli* (challenged groups: PC, HP, and EP) in 4 separate doses (1 mL each) over 2 d, with the following dosage concentrations: dose 1 (4.5 × 10^9^ CFU/pig), dose 2 (6.5 × 10^9^ CFU/pig), dose 3 (3.75 × 10^9^ CFU/pig), and dose 4 (4.75 × 10^9^ CFU/pig), for a final concentration of 2.0 × 10^10^ CFU/pig. The F18^+^*E. coli* of strain 2144 (O147: non-motile) was originally isolated from piglets with PWD and produced STa and STb (Sun et al., 2021). Pigs in the unchallenged group received a sterile saline solution. The cultures of the F18^+^*E. coli* strains were prepared following the standard laboratory protocol as previously reported (Duarte et al., 2020; Xu et al., 2022; Jang et al., 2023). Piglets and sows were not vaccinated against *E. coli* prior to this study. To minimize the possibility of cross-contamination, non-challenged pigs were housed in the same room separated by 2.10 m from challenged pigs. Solid barriers were also used to separate pens, and biosecurity protocols, including changing gloves and footwear, were followed between treatment areas. All daily procedures were conducted first with the non-challenged groups before interacting with the challenge group.

### GP and FS

Pigs and the feed disappearance were individually weighed at the end of each phase to determine GP parameters, including BW, average daily gain (**ADG**), average daily feed intake (**ADFI**), and gain-to-feed ratio (**G:F**). FS was recorded every day during the entire period based on a 1 to 5 scale, 1) very firm stool, 2) normal firm stool, 3) moderately loose stool, 4) loose, watery stool, and 5) very watery stool (Duarte et al., 2020).

### Sample collection

After day 28 of the study, all pigs were euthanized by exsanguination after a penetrating captive bolt to the head, to collect jejunal tissues (3 m away from the pyloric duodenal junction), rinsed with a saline solution (0.9%) to remove digesta content. The mid-jejunum was scraped to obtain intestinal mucosa placed in Eppendorf tubes (2 mL) and stored at −80 °C (after snap-freezing in liquid nitrogen, immediately after collection). A segment from the mid-jejunum was removed and placed in a 5 mL tube, immediately placed into liquid nitrogen, and then stored at −80 °C. Another segment of mid-jejunum tissue was removed, rinsed with a 0.9% saline solution, and stored in a 50 mL Falcon tube containing 10% buffered formaldehyde.

### Diversity and RA of jejunal mucosa-associated microbiota

Mucosa samples collected from the mid-jejunum were to determine the diversity and RA of jejunal mucosa-associated microbiota. The samples were sent to a commercial laboratory (Zymo Research, Irvine, CA) for 16S rRNA microbiome sequencing analysis. Utilizing the ZymoBIOMICS-96 MagBead DNA Kit (Zymo Research). The generation of the alpha diversity rare fraction plot and the amplicon sequence variant (**ASV**) table was executed using Qiime (version 1.9.1), with a depth of sequencing of 20,000 × sample preparation. The DADA2 pipeline was used to infer unique amplicon sequences that were inferred from the raw reads. Zymo Research Database, an internally curated 16S database, was utilized as a reference to assign taxonomy, using Uclust from QUIIME v.1.9.1. Alpha and beta diversity were evaluated using the website program, MicrobiomeAnalyst (QC, CA). The ASV data were transformed to RA. At each level, ASV information characterized by an RA of less than 0.5% and sequences that were not assigned were combined and collectively labeled as “Others” as previously described by Kim et al. (2019). Beta diversity was assessed using the Bray-Curtis distance method, as described by Deng et al. (2023).

### Relative mRNA expression of genes in jejunal tissue

Tissue samples obtained from the mid-jejunum were weighed (50 to 100 mg) and subjected to homogenization in 1 mL of TRIzol reagent (15-596-026, Invitrogen, Waltham, MA) utilizing the Bead Mill 24 homogenizer (Thermo Fisher Scientific Inc.). The homogenization process involved 2 cycles at 4.5 m/s for 30 s each, with a 20 s interval on ice between cycles, as previously described by Jang et al. (2023). Post-homogenization, the samples were centrifugated for 10 min at 12,000 × *g* at 4 °C. Following centrifugation, the resulting supernatant was transferred to a 1.5 mL centrifuge tube containing 200 µL of chloroform (Thermo Fisher Scientific Inc.) and gently vortexed for 1 min. The combined tubes were incubated at room temperature for 10 min, followed by another centrifugation for 15 min at 12,000 × *g* at 4 °C. The resulting supernatant was cautiously removed, leaving the pellet and dried within a fume hood for approximately 20 min until complete evaporation of the supernatant. The obtained RNA underwent quality and quantity assessment using spectrometry, as detailed by Jang et al. (2023). Subsequently, the extracted RNA was reverse transcribed into cDNA using a commercial kit (RevertAid First Strand cDNA Synthesis, Thermo Fisher Scientific Inc.), following the manufacturer’s instructions. For quantitative real-time polymerase chain reaction (**qPCR**), the CFX Connect Real-Time PCR Detection System (BioRad, Hercules, CA, USA) was employed, using Maxima SYBR Green/ROX qPCR Master Mix (Thermo Fisher Scientific Inc.) and oligonucleotide primers synthesized by Millipore Sigma (Burlington, MA). The genes evaluated were nod-like receptor 1 (NOD1); nod-like receptor 2 (NOD2); toll-like receptor 2 (**TLR2**); toll-like receptor 4 (**TLR4**); claudin (**CLD**); occludin (**OCC**); zonula occludens-1 (**ZO-1**). The primers are listed in Table 3. The relative expression of each gene was normalized using delta–delta–Ct method as described previously (Jang et al., 2023).

**Table 3.** Sequence of primers for microbial sensing and intestinal integrity in the jejunum of nursery pigs fed diets supplemented with ground herb-based phytobiotics^1^ and essential oil-based phytobiotics^2^

Gene^3^	Primer sequences (5′–3′)^4^	Accession number	Size
NOD1	F: AACACCGATCCAGTGAGCAG R: AAATGGTCTCGCCCTCCTTG	NM_001114277.1	230
NOD2	F: GTGCCTCCCCTCTAGACTCA R: ACGAACCAGGAAGCCAAGAG	NM_001105295.1	191
TLR2	F: GGGCTGCGTTCATTCATCAG R: CTGCAGAGGATGGATGGCAA	XM_005653576.3	132
TLR4	F: CGTGCAGGTGGTTCCTAACA R: GGTTTGTCTCAACGGCAACC	NM_001113039.2	326
CLD	F: AAACCGTGTGGGAACAACCA R: CACATGAAAATGGCTTCCCTC	NM_001244539.1	196
OCC	F: TCAGGTGCACCCTCCAGATT R: AGGAGGTGGACTTTCAAGAGG	XP_005672579.1	169
ZO-1	F: CAGAGACCAAGAGCCGTCC R: TGCTTCAAGACATGGTTGGC	XM_003480423.4	105

^1^Salcochek Pro (Ayurvet Limited).

^2^Liq-biotic (Ayurvet Limited).

^3^NOD1, nod-like receptor 1; NOD2, nod-like receptor 2; TLR2, toll-like receptor 2; TLR4, toll-like receptor 4; CLD, claudin; OCC, occludin; ZO-1, zonula occludens-1.

^4^F, forward; R, reverse.

### Inflammatory cytokines, humoral immune status, and oxidative stress parameters in the jejunum

Mucosa samples collected from the mid-jejunum were weighed (1 g) and placed in 1 mL of phosphate-buffered saline (**PBS**) on ice. Subsequently, the samples were homogenized using a tissue homogenizer (Tissuemiser; Thermo Fisher Scientific Inc.) the samples underwent centrifugation at 14,000 × *g* for 15 min, following the methodology described by Holanda and Kim (2021). Upon centrifugation, the supernatant was carefully extracted, divided into 5 aliquots, and stored at −80 °C for subsequent analyses.

The concentrations of total protein, malondialdehyde (**MDA**), protein carbonyl, immunoglobulin G (**IgG**), immunoglobulin A (**IgA**), tumor necrosis factor-alpha (**TNF-α**), interleukin-6 (**IL-6**), and interleukin-8 (**IL-8**), were determined using commercially available kits as per the provided instructions. Optical density (**OD**) values were measured using a plate reader (Synergy HT, BioTek Instruments, Winooski, VT) and analyzed with corresponding software (Gen5 Data Analysis Software, BioTek Instruments). Concentrations of each analyte were calculated by comparing the resulting OD values to the absorbance of standard curves following the guidelines provided in the manual.

To proceed with the laboratory analysis, the supernatant derived from the earlier homogenization of mucosal samples underwent a dilution step (1:60) in PBS to achieve a suitable concentration range (20 to 2,000 μg/mL). The determination of total protein concentration was carried out utilizing the Pierce BCA Protein Assay Kit (#23225, Thermo Fisher Scientific Inc.), following the methodology outlined by Holanda et al. (2020). Absorbance readings were taken at 562 nm, and the resulting total protein concentrations for each sample were employed for normalization in subsequent colorimetric assays. The measurement of MDA concentration in the mucosa was conducted using the OxiSelect TBARS MDA Quantitation Assay Kit (#STA-330, Cell Biolabs, Inc., San Diego, CA), following the protocol detailed by Moita et al. (2021). The working range for the standard is between 0.98 and 125 µM/L, with absorbance readings conducted at a wavelength of 532 nm. The MDA concentration was calculated using the standard and expressed as nmol/mg protein. For the quantification of protein carbonyl, the OxiSelect Protein Carbonyl ELISA Kit (#STA-310, Cell Biolabs, Inc.) was utilized in accordance with the methodology outlined by Moita et al. (2021). Supernatants were appropriately diluted with PBS to obtain a protein concentration of 10 µg/mL before measurement. The standard was prepared within the working range of 0.375 to 7.5 nmol/mg protein. All steps were performed in accordance with the provided manual, and absorbance readings were taken at a wavelength of 450 nm, with concentrations expressed as nmol/mg protein. The concentrations of IgG and IgA were determined using ELISA kits (E101-104 and E101-102, Bethyl Laboratories, Inc., Montgomery, TX) following the protocol outlined by Holanda et al. (2020). Mucosal sample supernatants were appropriately diluted with PBS to achieve dilution factors of 1:1,600 and 1:400 for IgG and IgA, respectively, ensuring the working range for measurement. Absorbance readings were taken at a wavelength of 450 nm, and concentrations were reported as µg/mg of protein. The TNF-α concentration was quantified using the Porcine TNF-α Immunoassay Kit (#PTA00, R&D Systems, Minneapolis, MN, USA), as detailed in previous work (Cheng et al., 2021). Absorbance readings at 450 nm, corrected at 570 nm, were used to determine the final TNF-α concentration, expressed as pg/mg protein. The concentration of IL-6 was determined by employing the Porcine IL-6 DuoSet ELISA kit (#DY686, R&D Systems) in accordance with the method outlined by Deng et al. (2023). Similarly, for the measurement of IL-8 concentration, the Porcine IL-8/CXCL8 DuoSet ELISA kit (#DY535, R&D Systems) was used following the procedure described in previous work (Jang and Kim, 2019). Prior to analysis, samples were diluted with reagent diluent at a 1:5 ratio. Absorbance readings were taken at 450 nm, with correction at 570 nm. All steps were executed following the manufacturer’s protocol.

### Intestinal morphology and cell proliferation (Ki-67^+^) in the crypt of the jejunum

Jejunal tissues were used for morphological evaluation. Following sampling, sections extracted from the mid-jejunum of each pig were fixed in a 10% formalin solution for a duration of 3 d. Subsequently, the samples were transversely cut into 2 sections, placed in a cassette, and immersed in a 70% ethanol solution. These prepared sample sections were then shipped to the University of North Carolina School of Medicine Lineberger Comprehensive Cancer Center (Chapel Hill, NC) for further processing. Formalin-fixed tissues were processed using a Leica ASP 6025s tissue processor, embedded in paraffin (Leica Paraplast), and sectioned at a thickness of 5 µm. Chromogenic Immunohistochemistry was performed on tissue slides using the Ventana Discovery automated staining platform (Roche). Antigen retrieval was carried out using Ventana’s CC1 solution (pH 8.5), followed by blocking, and incubation with a Ki-67 primary antibody (12202S, Cell Signaling Technology) diluted at 1:400. A secondary antibody (Discovery OmniMap anti-Rabbit HRP, Roche) was applied, and Ki-67^+^ cells were visualized with Discovery Chromo Maps DAB (Roche), followed by nuclear counterstaining with Hematoxylin II (Roche). Stained slides were dehydrated and the cover slipped with Cytoseal 60 (Epredia). A PC was included in the staining procedure. The evaluation of samples involved the use of an Olympus CX31 microscope (Lumenera Corporation, Ottawa, Canada) and the Infinity 2-2 digital CCD software. For each sample, images capturing 10 intact villi, and their associated crypts were obtained and measured, as previously detailed (Jang et al., 2020; Cheng et al., 2021). The measurements included villus height (**VH**) from the top to the junction of the villus and crypt, and crypt depth (**CD**) from the junction of the villus and crypt to the crypt’s bottom. The villus height to crypt depth (**VH:CD**) ratio was determined by dividing the measured VH by CD. In the Teledyne Lumenera INFINITY ANALYZE 7 software, the 10 images for each sample were imported, and the number of Ki-67^+^ cells in the crypt was counted. A single person conducted image collection and analysis of the intestinal morphology samples, and the averaged results of 10 measurements per pig were reported as a singular value per pig.

### Statistical analysis

Data was analyzed using Proc MIXED SAS 9.4 software (SAS Inc., Cary, NC, USA). Initial BW and sex were considered as blocks. The statistical model included dietary treatments as fixed effects and blocks as random effects. Experimental unit was the individually housed pig. A power test was conducted to determine the appropriate number of replications needed for the study to determine statistical significance for an expected mean difference of 11% to 12% at *P* < 0.05. Based on previous studies conducted with pigs with a similar genetic background in the same research facility (Duarte et al., 2020; Duarte and Kim, 2022; Jang et al., 2023), a coefficient of variation of 7.5% was utilized. The power of test (1—beta) at 95% the power analysis indicated an 80%, the minimum number of replications for each treatment was 10 (Aaron and Hays, 2004). A pre-determined contrast was utilized to determine the differences of least squares means between NC vs. PC treatments, to test the effect of challenge with F18^+^*E. coli*, and between PC vs. HP, and PC vs. EP treatments. The data related to diarrhea incidence were analyzed using the Proc Freq of SAS. The analysis of similarities (**ANOSIM**) was conducted to assess the beta diversity of mucosa-associated microbiota, with the results visualized using principal coordinate analysis (**PCoA**) based on Bray-Curtis distance. Statistical differences were considered significant with *P *< 0.05 and tendency with 0.05 ≤ *P* < 0.10.

## Results

### Diversity and RA of jejunal mucosa-associated microbiota

**Figure 1.**
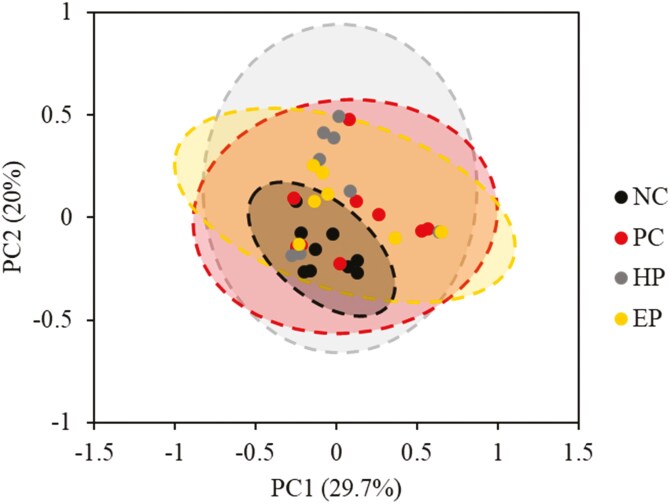
PCoA plot in the jejunal mucosa-associated microbiota at the species level in nursery pigs fed diets supplemented with ground herb-based phytobiotics and essential oil-based phytobiotics. NC, basal diet, non-challenged pigs; PC, basal diet, challenged with F18^+^*E. coli*; HP, basal diet + 1% ground herb-based phytobiotics (Salcochek Pro, Ayurvet Limited), challenged with F18^+^*E. coli*; EP: basal diet + 1% essential oil-based phytobiotics (Liq-biotic, Ayurvet Limited), challenged with F18^+^*E. coli*. The X-axis and Y-axis represent the principal component axes, with the percentages indicating the proportion of variation explained by each component. Points of different colors correspond to samples from different treatments (NC, PC, HP, and EP), and the closer the 2 points are, the more similar their species composition. The *P* value for Bray-Curtis for NC vs. PC: (*P* = 0.025); PC vs. HP (*P* = 0.458); PC vs. EP (*P* = 0.744).

There were no differences between PC and NC treatment for alpha diversity of jejunal mucosa-associated microbiota (Table 4). The HP treatment decreased (*P* < 0.05) alpha diversity of Chao1, Shannon, and Simpson when compared to PC treatment. The microbial community was visualized using PCoA based on Bray-Curtis distance, which confirmed that the PC changed (*P* < 0.05) microbiota composition in the jejunal mucosa of nursery pigs compared to the NC group (Figure 1). There were no significant differences in beta diversity of the jejunal mucosa-associated microbiota for HP and EP compared to PC treatment.

**Table 4.** Alpha diversity of jejunal mucosa-associated microbiota at species level in pigs fed diets supplemented with ground herb-based phytobiotics^1^ and essential oil-based phytobiotics^2^

		Treatment^3^				*P* value		
Item	NC	PC	HP	EP	SEM	NC vs. PC	PC vs. HP	PC vs. EP
Chao1	37	36	28	32	2.0	0.921	0.013	0.240
Shannon	1.5	1.7	1.1	1.2	0.2	0.557	0.021	0.581
Simpson	0.7	0.7	0.4	0.6	0.1	0.824	0.018	0.487

^1^Salcochek Pro (Ayurvet Limited).

^2^Liq-biotic (Ayurvet Limited).

^3^NC, basal diet, non-challenged pigs; PC, basal diet, challenged with F18^+^*E. coli*; HP, basal diet + 1% ground herb-based phytobiotics, challenged with F18^+^*E. coli*; EP, basal diet + 1% essential oil-based phytobiotics, challenged with F18^+^*E. coli*.

**Figure 2.**
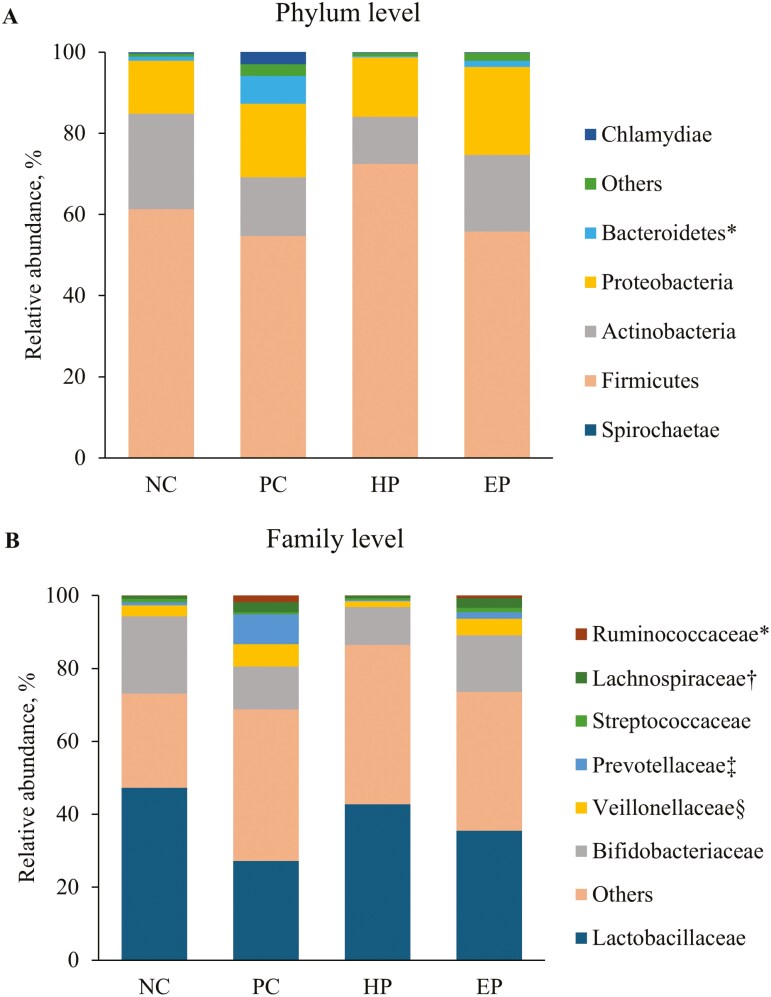
RA of jejunal mucosa-associated microbiota at (A) phylum and (B) genus level in pigs fed diets supplemented with ground herb-based phytobiotics and essential oil-based phytobiotics. Each pattern represents a particular bacterial phylum. Phylum sequences that did not achieve 0.5% within each phylum were combined as “Others”. NC, basal diet, non-challenged pigs; PC, basal diet, challenged with F18^+^*E. coli*; HP, basal diet + 1% ground herb-based phytobiotics (Salcochek Pro, Ayurvet Limited), challenged with F18^+^*E. coli*; EP: basal diet + 1% essential oil-based phytobiotics (Liq-biotic, Ayurvet Limited), challenged with F18^+^*E. coli*. (A). * Bacteroidetes, NC vs. PC: (*P *< 0.05), PC vs. HP: (*P *< 0.05), PC vs. EP (*P *= 0.057). (B) *Ruminococcaceae NC vs. PC: (*P *< 0.05), PC vs. HP: (*P *= 0.062), PC vs. EP (*P *= 0.238); †Lachnospiraceae NC vs. PC: (*P *< 0.05), PC vs. HP: (*P *< 0.05), PC vs. EP (*P *= 0.941); ‡Prevotellaceae NC vs. PC: (*P *< 0.05), PC vs. HP: (*P *< 0.05), PC vs. EP (*P *= 0.111); §Veillonellaceae NC vs. PC: (*P* = 0.133), PC vs. HP: (*P *< 0.05), PC vs. EP (*P *= 0.480).

At phylum level (Table 5), the PC treatment increased (*P* < 0.05) the RA of Bacteroidetes when compared to NC treatment (fig. 2A). The PC treatment tended to decrease (*P* = 0.085) the RA of Actinobacteria, whereas PC treatment tended to increase (*P* = 0.074) Spirochaetae when compared to NC treatment. The HP treatment decreased (*P* < 0.05) Bacteriodetes when compared to PC treatment, whereas EP treatment tended to reduce (*P* = 0.057) Bacteriodetes when compared to PC treatment.

**Table 5.** RA of jejunal mucosa-associated microbiota at the phylum level in pigs fed diets supplemented with ground herb-based phytobiotics^1^ and essential oil-based phytobiotics^2^

	Treatment^3^					*P* value		
Item	NC	PC	HP	EP	SEM	NC vs. PC	PC vs. HP	PC vs. EP
Firmicutes	61.29	54.56	72.47	55.66	8.15	0.534	0.125	0.927
Actinobacteria	23.52	14.46	11.55	18.91	3.87	0.085	0.593	0.433
Proteobacteria	13.06	18.11	14.63	21.61	8.94	0.669	0.781	0.788
Bacteroidetes	1.00	6.84	0.40	1.55	1.95	0.022	0.018	0.057
Chlamydiae	0.39	3.05	0.18	0.18	1.58	0.172	0.165	0.180
Spirochaetae	< 0.01	0.15	0.02	0.12	0.07	0.074	0.145	0.758
Others	0.74	2.83	0.75	1.97	0.70	0.029	0.040	0.401

^1^Salcochek Pro (Ayurvet Limited).

^2^Liq-biotic (Ayurvet Limited).

^3^NC, basal diet, non-challenged pigs; PC, basal diet, challenged with F18^+^*E. coli*; HP, basal diet + 1% ground herb-based phytobiotics, challenged with F18^+^*E. coli*; EP, basal diet + 1% essential oil-based phytobiotics, challenged with F18^+^*E. coli*.

At the family level (Table 6), the PC treatment increased (*P* < 0.05) Prevotellaceae, Lachnospiraceae, and Ruminococcaceae when compared to NC treatment (Figure 2B) and tended to decrease the RA of Lactobacillaceae (*P* = 0.092), and Bifidobacteriaceae (*P* = 0.050) when compared to NC treatment. The HP treatment decreased (*P* < 0.05) the RA of Veillonellaceae, Prevotellaceae, and Lachnospiraceae when compared to NC treatment, and tended to reduce (*P* = 0.062) RA of Ruminococcaceae when compared to PC treatment, whereas EP treatment tended to increase the RA of Streptococcaceae (*P* = 0.073) and Corynebacteriaceae (*P* = 0.074) when compared to PC treatment.

**Table 6.** RA of jejunal mucosa-associated microbiota at family level in pigs fed diets supplemented with ground herb-based phytobiotics^1^ and essential oil-based phytobiotics^2^

	Treatment^3^					*P* value		
Item	NC	PC	HP	EP	SEM	NC vs. PC	PC vs. HP	PC vs. EP
Lactobacillaceae	47.26	27.17	42.77	36.49	8.79	0.092	0.212	0.569
Bifidobacteriaceae	21.14	12.78	10.38	15.84	3.50	0.050	0.780	0.486
Staphylococcaceae	6.94	14.14	25.26	5.96	6.86	0.644	0.149	0.591
Helicobacteraceae	11.71	18.64	14.13	20.95	8.72	0.734	0.902	0.676
Veillonellaceae	2.99	6.10	1.59	4.54	1.54	0.133	0.043	0.480
Prevotellaceae	0.92	4.18	0.37	1.83	1.17	0.045	0.028	0.111
Coriobacteriaceae	1.15	2.24	0.93	2.11	0.61	0.179	0.132	0.884
Lachnospiraceae	0.82	2.75	0.61	2.67	0.69	0.041	0.033	0.941
Erysipelotrichaceae	0.68	0.70	1.02	1.44	0.37	0.970	0.527	0.170
Leuconostocaceae	0.94	0.95	0.19	0.91	0.63	0.999	0.386	0.966
Chlamydiaceae	0.39	0.47	0.18	0.18	0.21	0.765	0.332	0.263
Streptococcaceae	0.90	0.62	0.52	1.19	0.21	0.328	0.726	0.073
Corynebacteriaceae	0.86	0.24	0.12	1.15	0.34	0.180	0.803	0.074
Ruminococcaceae	0.09	1.84	0.09	0.71	0.72	0.049	0.062	0.238
Others	3.21	7.18	1.84	4.03	1.44	0.048	0.014	0.149

^1^Salcochek Pro (Ayurvet Limited).

^2^Liq-biotic (Ayurvet Limited).

^3^NC, basal diet, non-challenged pigs; PC, basal diet, challenged with F18^+^*E. coli*; HP, basal diet + 1% ground herb-based phytobiotics, challenged with F18^+^*E. coli*; EP, basal diet + 1% essential oil-based phytobiotics, challenged with F18^+^*E. coli*.

At the genus level (Table 7), the PC treatment tended to decrease the RA of *Lactobacillus* (*P* = 0.092) and *Bifidobacterium* (*P* = 0.050) when compared to NC treatment. The HP treatment tended to reduce the RA of *Megasphera* (*P* = 0.074) and *Dialister* (*P* = 0.052) when compared to PC treatment. The EP treatment increased (*P* < 0.05) *Syntrophococcus* when compared to PC treatment, whereas EP tended to increase *Streptococcus* (*P* = 0.076) and *Corynebacterium* (*P* = 0.098) when compared to PC treatment.

**Table 7.** RA of jejunal mucosa-associated microbiota at genus level in pigs fed diets supplemented with ground herb-based phytobiotics^1^ and essential oil-based phytobiotics^2^

	Treatment^3^					*P* value		
Item	NC	PC	HP	EP	SEM	NC vs. PC	PC vs. HP	PC vs. EP
*Lactobacillus*	47.24	27.12	42.76	34.25	8.80	0.092	0.211	0.581
*Bifidobacterium*	21.13	12.77	10.38	15.36	3.50	0.050	0.777	0.486
*Staphylococcus*	6.92	12.08	25.24	5.76	6.86	0.647	0.148	0.560
*Helicobacter*	11.71	15.84	14.13	20.95	8.72	0.734	0.902	0.676
*Megasphaera*	1.10	2.67	0.74	1.38	0.63	0.134	0.074	0.285
*Olsenella*	0.98	2.45	0.82	1.88	0.58	0.146	0.116	0.777
*Weissella*	0.94	0.95	0.19	4.15	1.41	0.999	0.703	0.131
*Mitsuokella*	1.11	1.10	0.53	1.04	0.39	0.976	0.187	0.899
*Chlamydia*	0.39	3.05	0.18	0.18	1.58	0.172	0.165	0.180
*Streptococcus*	0.90	0.62	0.52	1.18	0.21	0.324	0.728	0.076
*Syntrophococcus*	0.53	0.64	0.40	1.70	0.31	0.777	0.571	0.019
*Sharpea*	0.29	0.23	0.84	0.97	0.31	0.887	0.168	0.109
*Corynebacterium*	0.66	0.17	0.09	0.80	0.25	0.157	0.824	0.098
*Dialister*	0.54	0.64	0.10	0.44	0.19	0.701	0.052	0.483
*Selenomonas*	0.09	0.28	0.03	0.35	0.14	0.280	0.189	0.773
*Prevotella*	0.08	0.45	0.06	0.11	0.17	0.102	0.111	0.179
Others	5.39	18.94	2.99	9.50	4.73	0.020	0.011	0.135

^1^Salcochek Pro (Ayurvet Limited).

^2^Liq-biotic (Ayurvet Limited).

^3^NC, basal diet, non-challenged pigs; PC, basal diet, challenged with F18^+^*E. coli*; HP, basal diet + 1% ground herb-based phytobiotics, challenged with F18^+^*E. coli*; EP, basal diet + 1% essential oil-based phytobiotics, challenged with F18^+^*E. coli*.

At the species level (Table 8), the PC treatment decreased (*P* < 0.05) the RA of *Lactobacillus delbrueckii*, *Bifidobacterium dentium*, *Lactobacillus salivarius*, and *Bifidobacterium boum* when compared to NC treatment. The HP treatment increased (*P* < 0.05) the RA of *Staphylococcus saprophyticus* and *Staphylococcus cohnii-nepalensis* when compared to PC treatment and tended to decrease (*P* = 0.054) the RA of *Dialister succinatiphilus*. The EP treatment increased (*P* < 0.05) the RA of *Syntrophococcus* sp. when compared to PC treatment.

**Table 8.** RA of mucosa-associated microbiota at species level of pigs fed diets supplemented with ground herb-based phytobiotics^1^ and essential oil-based phytobiotics^2^

	Treatment^3^					*P* value		
Item	NC	PC	HP	EP	SEM	NC vs. PC	PC vs. HP	PC vs. EP
*Helicobacter rappini*	11.68	13.08	14.03	20.13	8.51	0.897	0.934	0.575
*Bifidobacterium thermacidophilum-thermophilum*	7.07	7.98	6.36	13.04	2.97	0.816	0.698	0.248
*Lactobacillus delbrueckii*	20.58	9.00	18.20	12.97	4.32	0.045	0.130	0.530
*Lactobacillus mucosae*	9.02	6.21	8.89	8.46	2.26	0.351	0.401	0.515
*Staphylococcus kloosii*	2.04	6.61	6.55	1.08	3.53	0.287	0.989	0.243
*Staphylococcus saprophyticus-xylosus*	1.67	1.98	4.11	2.95	1.35	0.863	0.266	0.694
*Bifidobacterium dentium*	10.06	2.11	2.51	0.04	1.84	0.001	0.853	0.339
*Staphylococcus saprophyticus*	1.35	0.93	4.24	1.31	0.99	0.748	0.023	0.794
*Staphylococcus cohnii-nepalensis*	0.73	1.27	9.81	0.18	2.86	0.887	0.040	0.794
*Bifidobacterium boum*	4.08	1.55	1.51	2.25	0.86	0.026	0.971	0.558
*Megashpaera* sp.	1.07	2.33	0.74	1.34	0.63	0.136	0.079	0.281
*Chlamydia muridarum*	0.39	3.05	0.18	0.18	1.58	0.172	0.165	0.180
*Lactobacillus salivarius*	1.79	0.33	0.79	0.71	0.60	0.004	0.530	0.611
*Olsenella profusa*	0.81	1.12	0.61	0.79	0.41	0.567	0.378	0.579
*Syntrophococcus* sp.	0.46	0.63	0.38	1.66	0.31	0.664	0.540	0.021
*Weissellat hailandensis*	0.65	0.86	0.17	2.28	0.73	0.838	0.538	0.223
*Sharpea azabuensis*	0.29	0.23	0.82	0.97	0.29	0.887	0.168	0.109
*Helicobacter equorum*	0.02	1.90	0.02	0.84	0.58	0.014	0.018	0.189
*Mitsuokella multacida*	0.34	0.62	0.44	0.32	0.28	0.276	0.511	0.291
								
*Dialister succinatiphilus*	0.53	0.63	0.10	0.44	0.19	0.702	0.054	0.495
*Lactobacillus* sp.	11.95	8.02	4.72	2.95	2.72	0.279	0.391	0.207
Others	13.08	28.94	14.38	24.80	6.08	0.056	0.095	0.639

^1^Salcochek Pro (Ayurvet Limited).

^2^Liq-biotic (Ayurvet Limited).

^3^NC, basal diet, non-challenged pigs; PC, basal diet, challenged with F18^+^*E. coli*; HP, basal diet + 1% ground herb-based phytobiotics, challenged with F18^+^*E. coli*; EP, basal diet + 1% essential oil-based phytobiotics, challenged with F18^+^*E. coli*.

### Relative mRNA expression of genes in jejunal tissue

The F18^+^*E. coli* challenge did not alter the expression of NOD1 and NOD2 between treatments (Table 9). Although no differences were observed in NOD1, NOD2, TLR2, CLD, and ZO-1 expression, the PC treatment tended to increase (*P* = 0.096) TLR4 expression when compared to NC treatment and increased (*P* < 0.05) OCC expression when compared to NC treatment.

**Table 9.** Relative gene expression of mid-jejunum markers of pigs fed diets supplemented with ground herb-based phytobiotics^1^ and essential oil-based phytobiotics^2^

Item^3^	Treatment^4^				SEM	*P* value		
	NC	PC	HP	EP		NC vs. PC	PC vs. HP	PC vs. EP
NOD1	1.104	1.058	1.322	0.947	0.196	0.870	0.349	0.691
NOD2	1.034	1.036	1.276	0.945	0.149	0.993	0.263	0.672
TLR2	1.047	0.845	0.788	0.684	0.133	0.289	0.764	0.399
TLR4	1.064	2.990	2.075	2.353	0.796	0.096	0.422	0.575
CLD	1.027	1.685	1.243	2.450	0.518	0.287	0.472	0.189
OCC	0.997	1.408	1.568	1.370	0.113	0.014	0.324	0.809
ZO-1	1.003	1.091	1.169	1.000	0.092	0.509	0.551	0.492

^1^Salcochek Pro (Ayurvet Limited).

^2^Liq-biotic (Ayurvet Limited).

^3^NOD1, Nucleotide-binding oligomerization domain 1; NOD2, Nucleotide-binding oligomerization domain; TLR2, Toll-like receptor 2; TLR4, Toll-like receptor 2; CLD, claudin; OCC, occludin; ZO-1, Zonula occludens-1.

^4^NC, basal diet, non-challenged pigs; PC, basal diet, challenged with F18^+^*E. coli*; HP, basal diet + 1% ground herb-based phytobiotics, challenged with F18^+^*E. coli*; EP, basal diet + 1% essential oil-based phytobiotics, challenged with F18^+^*E. coli*.

### Oxidative stress and immune parameters in the jejunal mucosa

The PC treatment showed no difference in inflammatory markers (TNF-α, IL-6, and IL-8), immune response markers (IgA and IgG), or oxidative markers (MDA and protein carbonyl) in mucosa samples from the mid-jejunum at 28 d post-challenge when compared to NC treatment (Table 10). However, the HP treatment tended to decrease (*P *= 0.053) protein carbonyl in mucosa samples from the mid-jejunum compared to the PC treatment at 28 d post-challenge.

**Table 10.** Oxidative damage products and immune response from mid-jejunal mucosa of pigs fed with ground herb-based phytobiotics^1^ and essential oil-based phytobiotics^2^

Item^3^	Treatment^4^				SEM	*P* value		
	NC	PC	HP	EP		NC vs. PC	PC vs. HP	PC vs. EP
Unit/mg protein								
IL-6, pg	29.7	27.0	36.2	23.9	4.7	0.642	0.153	0.652
IL-8, pg	1.45	1.42	1.21	1.55	0.22	0.931	0.494	0.693
TNFα, pg	3.67	2.85	3.97	3.72	0.57	0.334	0.159	0.260
IgA, μg	2.89	2.70	2.43	2.64	0.37	0.703	0.625	0.915
IgG, μg	2.14	1.88	2.47	2.17	0.45	0.686	0.353	0.648
MDA, μmol	0.42	0.41	0.45	0.40	0.09	0.911	0.716	0.970
Protein carbonyl, nmol	1.34	1.71	0.95	1.48	0.27	0.332	0.053	0.567

^1^Salcochek Pro (Ayurvet Limited).

^2^Liq-biotic (Ayurvet Limited).

^3^IL-6, interleukin-6, IL-8, interleukin-8; TNFα, tumor necrosis factor-alpha; IgA, immunoglobulin A; IgG, immunoglobulin G; MDA, malondialdehyde.

^4^NC, basal diet, non-challenged pigs; PC, basal diet, challenged with F18^+^*E. coli*; HP, basal diet + 1% ground herb-based phytobiotics, challenged with F18^+^*E. coli*; EP, basal diet + 1% essential oil-based phytobiotics, challenged with F18^+^*E. coli*.

### Intestinal morphology and cell proliferation (Ki-67^+^) in the crypt of the jejunum

The PC treatment decreased (*P* < 0.05) VH when compared to NC treatment (Table 11), whereas HP and EP were not different in VH when compared to PC treatment. There were no differences in CD between PC and NC treatments, but HP and EP decreased (*P* < 0.05) CD when compared to PC treatment. The VH:CD ratio was similar between NC and PC treatment, whereas HP and EP increased (*P* < 0.05) VH:CD ratio when compared to PC treatment. The PC treatment decreased (*P* < 0.05) cell proliferation number Ki-67^+^ when compared to NC treatment. The HP and EP decreased (*P* < 0.05) cell proliferation number Ki-67^+^ when compared to PC treatment.

**Table 11.** Intestinal morphology and cell proliferation in nursery pigs fed diets ground herb-based phytobiotics^1^ and essential oil-based phytobiotics^2^

Item^4^	Treatments^3^				SEM	*P* value		
	NC	PC	HP	EP		NC vs. PC	PC vs. HP	PC vs. EP
VH, µm	486	422	467	440	22.0	0.034	0.115	0.486
CD, µm	177	164	132	130	8.0	0.206	0.004	0.002
VH:CD	2.79	2.62	3.54	3.38	0.16	0.432	0.001	0.002
Ki-67^+^^5^, unit	53.9	67.4	48.3	53.4	3.2	0.005	0.001	0.004

^1^Salcochek Pro (Ayurvet Limited).

^2^Liq-biotic (Ayurvet Limited).

^3^NC, basal diet, non-challenged pigs; PC basal diet, challenged with F18^+^*E. coli*; HP, basal diet + 1% ground herb-based phytobiotics, challenged with F18^+^*E. coli*; EP, basal diet + 1% essential oil-based phytobiotics, challenged with F18^+^*E. coli*.

^4^VH, villus height; CD, crypt depth; VH:CD, Villus height to Crypt depth ratio.

^5^Ki-67^+^, cell proliferation rate.

### Fecal score

**Figure 3.**
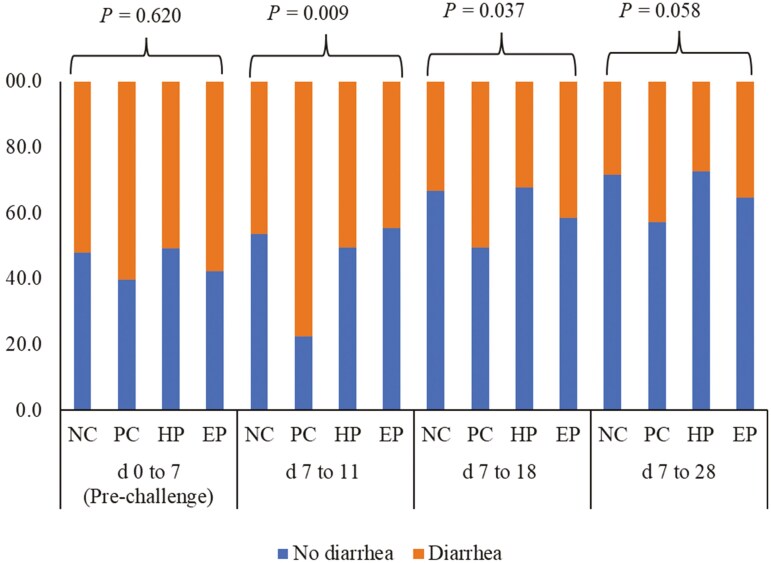
Incidence of diarrhea of pigs fed diets supplemented with ground herb-based phytobiotics and essential oil-based phytobiotics. NC, basal diet, non-challenged pigs; PC, basal diet, challenged with F18^+^*E. coli*; HP, basal diet + 1% ground herb-based phytobiotics (Salcochek Pro, Ayurvet Limited), challenged with F18^+^*E. coli*; EP: basal diet + 1% essential oil-based phytobiotics (Liq-biotic, Ayurvet Limited), challenged with F18^+^*E. coli*.

The incidence of diarrhea in the PC treatment was 30% higher compared to the HP and EP treatments during the period days 7 to 11 (Figure 3). The HP and EP treatment decreased (*P* < 0.05) FS when compared to PC treatment during the period days 7 to 11 and days 7 to 18.

### Growth performance

The PC treatment tended to have decreased BW at day 18 (*P* = 0.056) and day 21 (*P* = 0.059) when compared to NC treatment (Table 12). The PC treatment showed a reduced (*P* < 0.05) ADG in the last 2 phases of the study and reduced (*P* < 0.05) ADFI during the 3 phases of the study when compared to NC treatment. However, the PC treatment had no difference in G:F ratio when compared to NC treatment. Overall, ADG was still reduced by 25% and the final BW was still decreased by 2.4 kg in PC treatment when compared to NC treatment. As regards phytobiotics, results show that during the last periods post-challenge, there were no differences for HP and EP treatments for BW, ADG, and, consequently, G:F ratio, when compared to PC treatment.

**Table 12.** GP of pigs fed diets supplemented with ground herb-based phytobiotics^1^ and essential oil-based phytobiotics^2^

	Treatment^3^				*P* value			
Item	NC	PC	HP	EP	SEM	NC vs. PC	PC vs. HP	PC vs. EP
BW, kg								
Day 0	6.42	6.41	6.43	6.45	0.13	0.947	0.876	0.824
Day 7	6.37	6.31	6.27	6.37	0.18	0.805	0.855	0.824
Day 11	7.27	6.72	6.67	6.79	0.29	0.196	0.893	0.863
Day 18	10.58	9.18	9.10	9.36	0.50	0.056	0.906	0.807
Day 21	12.22	10.66	10.10	10.82	0.56	0.059	0.494	0.836
Day 28	16.07	13.67	12.72	13.87	0.72	0.024	0.354	0.851
ADG, g/d								
Days 0 to 7 (pre challenge)	-6	-15	-24	-11	17	0.732	0.719	0.881
Days 7 to 28	462	351	307	357	28	0.009	0.287	0.872
Days 0 to 11 (phase1)	77	28	21	31	23	0.143	0.815	0.929
Days 11 to 21 (phase 2)	495	393	344	403	33	0.034	0.290	0.840
Days 21 to 28 (phase 3)	551	431	374	435	40	0.044	0.322	0.946
Days 0 to 28 (overall)	345	260	225	265	24	0.017	0.308	0.874
ADFI, g/d								
Days 0 to 7 (pre challenge)	69	55	45	66	13	0.475	0.589	0.562
Days 7 to 28	730	539	477	522	38	0.001	0.254	0.743
Days 0 to 11 (phase1)	129	87	82	96	17	0.095	0.838	0.699
Days 11 to 21 (phase 2)	648	499	456	479	41	0.015	0.463	0.737
Days 21 to 28 (phase 3)	1,038	781	668	780	58	0.004	0.176	0.989
Days 0 to 28 (overall)	565	418	364	408	30	0.002	0.218	0.807
G:F								
Days 0 to 7 (pre challenge)	-0.21	-2.70	-2.39	-1.77	2.17	0.422	0.105	0.764
Days 7 to 28	0.63	0.64	0.65	0.68	0.03	0.827	0.846	0.299
Days 0 to 11 (phase1)	0.57	0.32	0.17	0.38	0.75	0.118	0.245	0.190
Days 11 to 21 (phase 2)	0.76	0.79	0.76	0.86	0.03	0.542	0.498	0.114
Days 21 to 28 (phase 3)	0.53	0.55	0.57	0.56	0.05	0.739	0.807	0.885
Days 0 to 28 (overall)	0.61	0.61	0.62	0.64	0.03	0.869	0.738	0.428

^1^Salcochek Pro (Ayurvet Limited).

^2^Liq-biotic (Ayurvet Limited).

^3^NC, basal diet, non-challenged pigs; PC, basal diet, challenged with F18^+^*E. coli*; HP, basal diet + 1% ground herb-based phytobiotics, challenged with F18^+^*E. coli*; EP, basal diet + 1% essential oil-based phytobiotics, challenged with F18^+^*E. coli*.

## Discussion

This study provides insights into how ground herb-based phytobiotics and essential oil-based phytobiotics differently influence intestinal health in nursery pigs challenged with F18^+^*E. coli*. Specifically, the findings demonstrate how these phytobiotics mitigate disruption to the jejunal mucosa-associated microbiota and support intestinal morphology, offering practical applications for improving intestinal health and performance in pig production.

Intestinal health is crucial as it is the primary site of digestion, nutrient absorption, and defense against pathogens, playing a key role in stress response in nursery pigs (Tang et al., 2022). In the present study, the F18^+^*E. coli* challenge induced jejunal mucosal-associated microbiota disruption and compromised intestinal morphology, evidenced by altered VH, CD, and tight junction protein expression (OCC) consistent with previous reports (Duarte et al., 2023; Jang et al., 2023). These changes were accompanied by disruption to the jejunal mucosa-associated microbiota, including reduced RA of Actinobacteria, known producers of antimicrobial agents (Barka et al., 2016), and increased susceptibility to intestinal inflammation and epithelial damage (Xu et al., 2023). Additionally, it was observed that F18^+^*E. coli* tended to reduce RA of *Lactobacillus*, which inhibits pathogenic bacteria through the production of lactic acid, antimicrobial peptides, and various metabolites. These actions contribute to improved GP and influence microbiota composition as well as immune indices in pigs (Pringsulaka et al., 2015; Zhang et al., 2023). Elevated TLR4 levels in challenged pigs reflected an active inflammatory response to the pathogen, consistent with its role in recognizing bacterial endotoxins like lipopolysaccharides from Gram-negative bacteria and activating innate immune signaling pathways (Sabroe et al., 2008; Hu et al., 2014). This aligns with studies showing that bacterial recognition by TLR4 can upregulate inflammatory responses (Chen et al., 2013).

Both phytobiotics mitigated these adverse effects, but through different mechanisms. The HP reduced microbial diversity, as observed by lower Chao1, Shannon, and Simpson indices, potentially limiting the proliferation of pathogenic bacteria (Xia and Sun, 2023). The bioactive compounds in HP, such as alkaloids and phenolics, can bind to bacterial DNA and RNA, altering their structure and disrupting replication, transcription, and protein synthesis of harmful bacteria (Jin et al., 2010). Additionally, alkaloids can interfere with bacterial metabolic pathways, leading to cell death of harmful bacteria (Du et al., 2020). Interestingly, HP treatment tended to reduce the RA of *Megasphaera* and *Dialister*, which are known to produce short-chain fatty acids or to modulate the host’s immune system and produce propionate (Yoshikawa et al., 2018; Sakamoto et al., 2022), this reduction of *Megasphaera* could suggest that HP has the ability to selectively modulate jejunal mucosal-associated microbiota population. Furthermore, the impact of oxidative stress on intestinal health was also considered in the present study, using oxidative damage markers such as MDA and protein carbonyl. These markers serve as immediate indicators of oxidative stress, reflecting tissue damage and impaired function (Dalle-Donne et al., 2003; Cordiano et al., 2023). The HP demonstrated the potential to reduce oxidative stress, as reflected by a trend toward decreased protein carbonyl levels. This likely contributed to preserving tight junction integrity by reducing proteins and DNA damage (Dalle-Donne et al., 2003; Mateos and Bravo, 2007). The antioxidant and anti-inflammatory properties of flavonoids and tannins in HP could have further protected against epithelial damage and promoted intestinal recovery (Chandra et al., 2016; Tong et al., 2022). In contrast, the EP phytobiotics positively modulated jejunal mucosa-associated microbiota, by exerting jejunal conditions to shift the RA of beneficial bacteria such as *Syntrophococcus* and *Corynebacterium*, whereas reducing pathogenic populations such as Bacteroidetes, which is related to a lower capacity of the host to obtain energy from ingested feed and a lower capacity to store fat (Guo et al., 2008). Although the study did not directly assess the antimicrobial properties of the phytobiotics, changes in microbiota composition suggest mechanisms involving bacterial membrane disruption and inhibition of biomolecule synthesis (Jin et al., 2010; Valenzuela-Grijalva et al., 2017; Chen et al., 2020). The bioactive compounds in EP, such as thymol and eugenol, are related to disrupting the lipid fraction of bacterial membranes, altering membrane permeability, and causing leakage of intracellular materials (Trombetta et al., 2005; Guimarães et al., 2019). Similarly, although EP also contains antioxidant compounds like thymol and eugenol (Nagoor Meeran et al., 2017; da Silva et al., 2018), its lipid-focused antioxidant mechanism may be less effective in reducing overall oxidative stress compared to the broader activity of tannins and flavonoids in HP (Nagababu et al., 2010; Archana et al., 2011).

**Figure 4.**
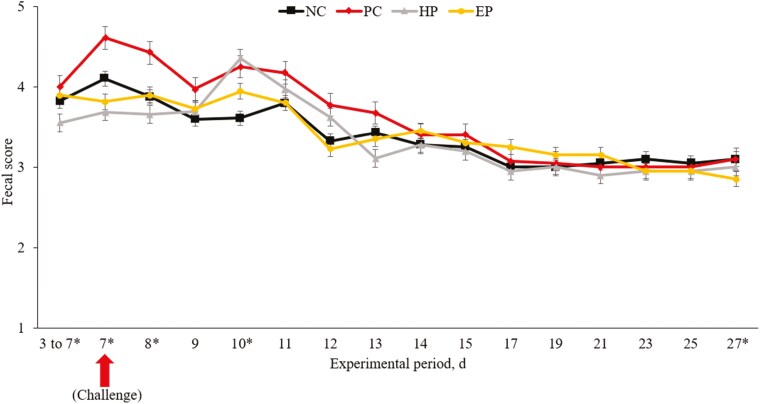
The FS of pigs fed diets supplemented with ground herb-based phytobiotics and essential oil-based phytobiotics. NC, basal diet, non-challenged pigs; PC, basal diet, challenged with F18^+^*E. coli*; HP, basal diet + 1% ground herb-based phytobiotics (Salcochek Pro, Ayurvet Limited), challenged with F18^+^*E. coli*; EP: basal diet + 1% essential oil-based phytobiotics (Liq-biotic, Ayurvet Limited), challenged with F18^+^*E. coli*. * days 3 to 7, NC vs. PC: (*P *= 0.259), PC vs. HP: (*P *= 0.007), PC vs. EP: (*P *= 0.527); day 7, NC vs. PC: (*P *= 0.238), PC vs. HP: (*P *= 0.045), PC vs. EP: (*P *= 0.080); day 8, NC vs. PC: (*P *= 0.061), PC vs. HP: (*P *= 0.018), PC vs. EP: (*P *= 0.527); day 10, NC vs. PC: (*P *= 0.029), PC vs. HP: (*P *= 0.751), PC vs. EP: (*P *= 0.362); day 27, NC vs. PC: (*P *= 1.000), PC vs. HP: (*P *= 0.385), PC vs. EP: (*P *= 0.035).

Despite differences in the mechanisms of action, the morphological changes observed in this study further highlight the protective effects of phytobiotics. The F18^+^*E. coli* challenge caused villus atrophy and crypt hyperplasia, as evidenced by reduced VH and increased CD in challenged pigs. However, supplementation with both types of phytobiotics improved intestinal morphology, as indicated by higher VH:CD ratios and reduced CD (Du et al., 2016; Chen et al., 2019). These results are supported by reduced Ki-67^+^ cells in phytobiotic-treated pigs, suggesting decreased cellular turnover and the presence of mature enterocytes (Paiva et al., 2014; Miller et al., 2018). The bioactive compounds in ground herb-based phytobiotics, such as alkaloids, flavonoids, and tannins, may promote epithelial regeneration and support intestinal structure (Peng et al., 2019; Pei et al., 2020; Toschi et al., 2022), while thymol and eugenol in essential oil-based phytobiotics also likely contribute to maintaining intestinal integrity (Rocha-Caldas et al., 2015; Hui et al., 2020). These improvements in intestinal morphology were accompanied by better FS, indicating reduced diarrhea severity (Figure 4). Whereas HP demonstrated prolonged reductions in diarrhea, likely due to their bioactive compounds’ ability to regulate gastrointestinal motility and electrolyte balance (Teke et al., 2010; Rahman et al., 2013; Damissie et al., 2023). The EP was more effective in the early post-challenge period. Though GP was not a primary focus of this study, the F18^+^*E. coli* challenge negatively impacted feed intake and growth during the initial postweaning period. This aligns with previous findings that suggest feed consumption is reduced under pathogenic stress (Li et al., 2019).

In conclusion, this study highlights the negative impacts of F18^+^*E. coli* on the jejunal mucosa-associated microbiota, immune responses, and intestinal morphology in nursery pigs. Both ground herb-based phytobiotics and essential oil-based phytobiotics influenced the composition of jejunal mucosa-associated microbiota by decreasing harmful bacteria, enhancing VH to crypt ratio, and promoting mucosal recovery through increased cell renewal and reducing FS in the first 2 wk post-challenge. However, the composition of ground herb-based phytobiotics, containing alkaloids, coumarins, flavonoids, saponins, and tannins, reduced oxidative stress, as shown by the tendency to decreased protein carbonyl levels in the jejunal mucosa. The distinct properties of herb-based and essential oil-based phytobiotics underscore their potential as natural interventions for improving intestinal health during *E. coli* challenges, and understanding their impacts is key to optimizing strategies for pig well-being.

## Abbreviations

ADFI: average daily feed intake
ADG: average daily gain
BW: body weight
G:F: gain to feed ratio
IgA: immunoglobulin A
IgG: immunoglobulin G
IL-6: interleukin-6
IL-8: interleukin-8
MDA: malondialdehyde
NOD1: nucleotide-binding oligomerization domain containing 1
NOD2: nucleotide-binding oligomerization domain containing 2
OCC: occludin
PWD: postweaning diarrhea
Sta: heat-stable toxin A
Stb: heat-stable toxin B
TLR2: toll-like receptor 2
TLR4: toll-like receptor 4
TNF-α: tumor necrosis factor-alpha
VH: villus height
VH:CD: villus height to crypt depth ratio
ZO-1: zonula occludens-1
